# Born to Fear the Machine? Genetic and Environmental Influences on Negative Attitudes toward AI Agents

**DOI:** 10.1002/advs.202506262

**Published:** 2025-06-23

**Authors:** Xiaojiayu Tan, Yue He, Yuan Zhou, Xinying Li, Qingwen Ding, Yikai Tang, Yu L. L. Luo, Ruolei Gu

**Affiliations:** ^1^ State Key Laboratory of Cognitive Science and Mental Health Institute of Psychology Chinese Academy of Sciences Beijing 100101 China; ^2^ Department of Psychology University of Chinese Academy of Sciences Beijing 100049 China; ^3^ Department of Psychology University of Toronto Toronto ON M5S 1A1 Canada; ^4^ Vector Institute Toronto ON M5G 1M1 Canada

**Keywords:** AI agents, attitude toward AI, moral preferences, twin study, victim sensitivity

## Abstract

Despite the rapid development of artificial intelligence (AI) agents, substantial individual differences in public acceptance persist. To explain the difference in attitudes toward AI agents, existing research has primarily focused on environmental factors. However, evolutionary psychology research suggests that the mechanism of outgroup rejection has a genetic basis, highlighting the need to explore the potential genetic underpinnings of negative attitudes toward AI agents as an outgroup in human society. This study examines the genetic basis of negative attitudes toward AI agents and their relationship with related personality traits, using a twin study design to assess negative attitudes toward AI agents, victim sensitivity, and moral preferences. Univariate genetic analyses revealed significant heritability of these negative attitudes. Bivariate analyses further identify shared genetic influences between victim sensitivity and personal‐level fear and wariness toward robots. Similarly, a shared genetic basis is observed between the moral preferences concerning authority and sociotechnical blindness anxiety toward AI agents. These findings extend the understanding of social cognition in AI agents by emphasizing the role of genetic factors in shaping attitudes toward them. Moreover, they provide new insights for enhancing public acceptance of AI agents and optimizing human‐machine interactions.

## Graphical abstract

Why do some people fear or distrust AI agents more than others? This twin study shows that negative attitudes toward AI may partly stem from genetic factors, linking them to traits like victim sensitivity and moral beliefs. These findings show that it is not only nurture but also nature that shapes our views of AI, with implications for future human–AI relations.

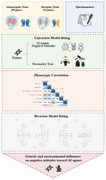

## 1 Introduction

In recent years, artificial intelligence (AI) agents have been rapidly penetrating various social domains, including healthcare, education, and daily services, profoundly reshaping human society and altering human behavior and lifestyle.^[^ ^1^, ^2^, ^3^ ^]^ AI agents refer to intelligent entities capable of perceiving their environment and influencing it through actions, encompassing both physical forms (e.g., robots) and virtual forms (e.g., voice assistants, intelligent software platforms).^[^ ^4^, ^5^, ^6^ ^]^ With the widespread adoption of AI agents, significant changes have occurred in work patterns, social interactions, and information access.^[^ ^7^ ^]^ However, public attitudes toward AI agents are not entirely positive. For example, research suggests that people generally hold more negative views toward robots, particularly in contexts involving high autonomy and social interaction.^[^ ^8^, ^9^, ^10^ ^]^ This negative perception primarily stems from three concerns. First, in fields like healthcare and economic decision‐making, although AI agents assist professionals by providing diagnoses and recommendations, their widespread adoption raises concerns about marginalization, anxiety over the declining value of human labor and job instability.^[^ ^11^, ^12^, ^13^ ^]^ Second, influenced by science fiction and media portrayals, some individuals fear that highly intelligent agents may develop autonomous decision‐making capabilities or even threaten human survival, exacerbating fears about risks associated with AI agents.^[^ ^11^, ^14^ ^]^ Finally, the appearance and behavior of AI agents may trigger the “uncanny valley effect,” wherein humanoid entities that closely but imperfectly resemble humans evoke discomfort due to perceived low social affinity.^[^ ^15^, ^16^, ^17^ ^]^ These negative attitudes not only affect individuals' acceptance of AI agents but may also hinder their widespread adoption and technological advancements innovation.^[^ ^18^, ^19^, ^20^ ^]^

Existing research on negative attitudes toward AI agents has primarily focused on sociocultural factors, generally attributing them to environmental experiences.^[^ ^21^, ^22^ ^]^ However, these explanations often overlook the potential role of genetic influences.^[^ ^23^, ^24^ ^]^ This neglect of genetic mechanisms may weaken the explanatory power of existing theories. Evolutionary psychology emphasizes that human behavioral tendencies are shaped by long‐term interactions between genetic and environmental factors.^[^ ^25^, ^26^, ^27^ ^]^ With the rapid development of AI agents, many people have begun to perceive them as a potential threat, a perception that may activate psychological defense mechanisms shaped through evolution. In this context, the Threat Management Theory (TMT) further proposes that humans have evolved adaptive defense mechanisms.^[^ ^28^ ^]^ When individuals encounter agents exhibiting outgroup characteristics, they tend to perceive either realistic or symbolic threats, influencing their attitudes and triggering automatic rejection responses.^[^ ^29^, ^30^ ^]^ Specifically, perceived realistic threats involve potential harm to personal or group resources and economic interests, whereas symbolic threats refer to concerns that outgroup members may introduce different cultural norms, challenging existing social orders.^[^ ^29^, ^31^, ^32^ ^]^ While such defensive strategies following the perception of threat were beneficial in prehistoric societies for minimizing risks related to pathogen transmission and resource competition,^[^ ^33^, ^34^ ^]^ in the AI era, they may lead to rejection of AI agents. Given that group‐based exclusion tendencies are heritable,^[^ ^35^, ^36^ ^]^ it is plausible that negative attitudes toward AI agents are also shaped by genetic‐neural mechanisms, rather than solely by environmental experiences.

Behavioral genetics examines the origins of individual differences in a behavior and the origins of correlations between different behaviors, by assessing the relative contributions of genetic, environmental, and gene‐environment interaction effects.^[^ ^37^, ^38^, ^39^ ^]^ From the perspective of behavioral genetics, certain personality traits—such as victim sensitivity (related to fairness in resource distribution) and moral preferences (associated with social norm) —have been shown to have substantial genetic bases.^[^ ^40^, ^41^, ^42^, ^43^ ^]^ These traits also significantly influence individuals’ attitudes toward AI agents. Specifically, victim sensitivity, an evolved vigilance mechanism against injustice, affects one's risk perception that the new outgroup members may damage the interests of the original group members,^[^ ^44^, ^45^ ^]^ whereas moral preferences reinforce adherence to social norms, affecting an individual's receptiveness to new outgroup members.^[^ ^46^, ^47^ ^]^ This study aims to investigate whether genetic factors determine or influence personality traits such as victim sensitivity and moral preferences, which in turn increase individuals’ susceptibility to negative attitudes toward AI agents. In other words, we examine whether individuals who are inherently more sensitive to resource distribution and social order are more likely to hold negative attitudes toward AI agents.

Twin studies, a classic method in the field of behavioral genetics, estimate genetic and environmental influences by comparing intrapair similarities between monozygotic and dizygotic twins using measures such as intraclass correlation coefficients.^[^ ^48^, ^49^, ^50^ ^]^ Given the above considerations, this study employs a twin study methodology to explore the genetic basis of negative attitudes toward AI agents and analyze the heritability of its correlation with victim sensitivity and moral preferences. From a theoretical perspective, this research represents the first attempt to examine the genetic mechanisms underlying negative attitudes toward AI agents from a behavioral genetics’ lens, expanding the existing framework of AI attitude research. By leveraging twin studies, we may improve previous neglect of genetic factors and provide new insights into interdisciplinary research between psychology and genetics.

The remainder of this paper is organized as follows: Section 2 reviews existing studies on negative attitudes toward AI agents, victim sensitivity, and moral preferences, identifying research gaps and supporting theoretical hypotheses. Section 3 presents the research hypotheses. Sections 4 and 5 outline the research methods and results, covering participant characteristics, questionnaire design, data collection, and analysis. Section 6 discusses the summary of key findings, the theoretical and practical contributions of the study, and Section 7 concludes with the research limitations, and directions for future work.

## 2 Literature Review

### 2.1 Dimensions of Negative Attitudes Toward AI Agents

People's negative attitudes toward AI agents include anxiety regarding these agents, fear and wariness toward them,^[^ ^51^ ^]^ as well as low perceived affinity toward them.^[^ ^11^, ^16^, ^52^, ^53^ ^]^ These attitudes often stem from uncertainties about the widespread application of AI agents, including its social impact, ethical concerns, and changes in human‐technology interaction patterns.^[^ ^11^, ^17^, ^54^ ^]^

#### 2.1.1 Fear and Wariness Toward AI Agents

Negative attitudes toward AI agents primarily involve two dimensions: cognition (negative perceptions of robots) and emotion (negative feelings toward robots).^[^ ^55^ ^]^ Research suggests that people exhibit different concerns and defensive responses toward the potential impact of AI agents at the personal and societal levels.^[^ ^56^, ^57^ ^]^

At the personal level, fear is often an instinctive reaction. Individuals may feel nervous or uncomfortable when interacting with robots, even without a specific reason. At the societal level, fear and wariness are more likely to arise from considerations of the broader impact of AI agents, such as concerns about the influence of autonomous driving systems on public transportation safety.^[^ ^55^ ^]^ Moreover, acceptance of AI agents varies across individual and societal levels depending on cultural backgrounds. For instance, in Japan, caregiving for the elderly and disabled is often viewed as a personal responsibility rather than a societal role to be fulfilled by robots. In contrast, Europeans tend to emphasize the societal contributions of robots but are less willing to involve them in their private lives.^[^ ^58^ ^]^ Additionally, research has found that students in AI‐related fields tend to hold more positive attitudes toward the impact of AI agents on their personal career development compared to their broader effects on societal progress,^[^ ^59^ ^]^ which further supports the dissociation between personal‐ and societal‐level attitudes toward AI.

Therefore, when studying fears toward AI agents, it is essential to differentiate between its impact on the personal and societal levels. Koverola et al.^[^ ^55^ ^]^ defined fear and wariness toward AI agents at the individual level as including aspects such as “robots are frightening,” “using robots in work environments is uncomfortable,” “feeling nervous when interacting with robots,” and “worrying that robots may fail to accurately understand commands.” At the societal level, their definition encompasses concerns about issues such as “robots may lead to human job losses,” “reducing human interactions,” “society becoming overly dependent on robots,” and “the need for strict regulation of robots.” In short, fear and wariness toward AI agents are multifaceted, encompassing both personal and societal levels. To gain a comprehensive understanding of their underlying mechanisms, it is essential to examine these attitudes from both personal and societal perspectives and to further explore the potential role of genetic influences.

#### 2.1.2 Anxiety Toward AI Agents

Anxiety toward AI agents refers to the concern in response to the rapid development and widespread adoption of AI agents.^[^ ^60^ ^]^ This anxiety encompasses multiple aspects, including concerns about job security, privacy invasion, information security risks, loss of human control over intelligent systems, and issues related to misinformation and algorithmic biases introduced by AI agents.^[^ ^61^, ^62^ ^]^ These aspects reflect people's psychological responses to the uncertainties and potential risks posed by the deep integration of intelligent technology into various social domains.^[^ ^60^, ^63^, ^64^ ^]^ Wang et al.^[^ ^61^ ^]^ categorized the anxiety toward AI agents into four major dimensions:

First, “job replacement anxiety” refers to the negative effects of AI agents on business life. Empirical evidence of systematic individual differences in job replacement anxiety comes from findings that employees who are proficient in cognitive tasks tend to be more concerned about being replaced by AI compared to those who excel in affective tasks.^[^ ^65^ ^]^ Second, “sociotechnical blindness” refers to fears and concerns about the potential loss of control over AI technologies or products. This includes worries about AI malfunctioning, being misused, causing unforeseen problems, or leading to autonomous behaviors that may escape human oversight.^[^ ^66^ ^]^ Third, “configuration anxiety” refers to fear or unease toward humanoid robots—robots whose appearance and movements closely resemble those of humans but exhibit subtle differences and whose internal mechanisms remain opaque—evoking negative emotions such as anxiety and discomfort.^[^ ^62^, ^67^ ^]^ And fourth, “learning anxiety” refers to anxiety regarding learning AI technologies and using AI agents. Older adults and digitally marginalized groups are particularly prone to this form of anxiety.^[^ ^68^, ^69^ ^]^ For example, researchers found that older participants expressed significant anxiety about adopting AI‐based technologies due to unfamiliarity with the technology and fears of making mistakes.^[^ ^70^ ^]^

The above framework suggests that people may experience various forms of anxiety toward AI agents for different reasons. It is therefore crucial to consider whether there is a genetic predisposition to anxiety toward AI agents and to examine this issue from multiple dimensions.

#### 2.1.3 Low Perceived Affinity Toward AI Agents

According to the Uncanny Valley Theory,^[^ ^15^ ^]^ when AI agents' appearance and behavior closely resemble humans but fall short of being completely realistic, individuals’ perceived affinity toward them drop sharply, leading to discomfort, aversion, or even fear.^[^ ^71^, ^72^ ^]^ Research indicates that when individuals perceive low affinity toward AI agents, their acceptance levels decrease accordingly, often accompanied by stronger algorithmic resistance and distrust of technology.^[^ ^16^, ^73^ ^]^ Thus, low perceived affinity is not only a critical indicator for assessing whether an AI agent falls into the “uncanny valley” but also directly influences people's overall attitudes toward AI agents and its societal adaptability.^[^ ^74^, ^75^ ^]^ To further understand individual differences in attitudes toward AI agents, this study also investigates whether low perceived affinity toward AI agents has a genetic basis.

### 2.2 The Relationship Between Negative Attitudes Toward AI Agents and Personality Traits

There are significant individual differences in people's attitudes toward AI technological changes.^[^ ^41^ ^]^ Two key personality traits associated with these differences are victim sensitivity (a heightened awareness of unfair resource distribution) and moral preferences (individual tendencies to endorse or prioritize specific moral values or foundations).^[^ ^31^, ^76^, ^77^ ^]^ Victim sensitivity enhances defensive responses to redistributions of benefits through a loss‐aversion mechanism.^[^ ^78^, ^79^ ^]^ Meanwhile, moral preferences primarily strengthen resistance to deviations from traditional norms.^[^ ^80^, ^81^ ^]^

#### 2.2.1 Victim Sensitivity and Negative Attitudes Toward AI Agents

Victim sensitivity is a personality trait with a genetic basis.^[^ ^40^, ^43^ ^]^ Initially used to assess individuals’ motivation for justice, this concept was later expanded to represent one of the four perspectives of injustice sensitivity (observer, beneficiary, victim, and perpetrator).^[^ ^82^ ^]^ Compared to other three perspectives, individuals with high victim sensitivity are particularly alert to external threats and deprivation and tend to perceive others as untrustworthy or potentially harmful.^[^ ^83^ ^]^ Such individuals often hold more negative attitudes toward new interaction partners and adopt stronger self‐protection strategies to reduce the risk of exploitation.^[^ ^78^, ^79^ ^]^ Meanwhile, research has shown that victim sensitivity has a significant genetic basis. For example, a biometric study of 1220 twin pairs by Eftedal et al.^[^ ^40^ ^]^ found that victim sensitivity is influenced by broad genetic strategies related to cooperation and dominance.

Individuals with high victim sensitivity are more likely to reject outgroup members and have greater difficulties in accepting new social interactions than those with lower victim sensitivity.^[^ ^84^, ^85^ ^]^ Some studies have explored the relationship between victim sensitivity and attitudes toward AI. For instance, Harjunen et al.^[^ ^45^ ^]^ found that in human‐computer interaction (HCI) contexts, individuals with high victim sensitivity were less likely to comply with virtual agents. Additionally, research suggests that people who are more sensitive to potential threats tend to have more negative attitudes toward AI and are more likely to resist its social applications.^[^ ^44^ ^]^ This tendency becomes more pronounced when individuals perceive an increased risk of exploitation or believe that in‐group goodwill may be taken advantage of by out‐groups.^[^ ^82^, ^83^, ^86^ ^]^ Based on this, the present study hypothesizes that victim sensitivity mediates negative attitudes toward AI agents, meaning that individuals with higher victim sensitivity are more likely to hold negative views toward AI agents.

#### 2.2.2 Moral Preferences and Negative Attitudes Toward AI Agents

Moral preferences, as a personality trait closely related to social norms, hierarchical structures, and intergroup ethical customs,^[^ ^87^, ^88^, ^89^ ^]^ are particularly important when assessing people's negative attitudes toward AI agents, especially when those agents are considered as out‐group members entering human society. Social identity theory and prior research on AI ethics suggest that AI agents often lie outside traditional human social categories,^[^ ^90^, ^91^, ^92^, ^93^, ^94^, ^95^ ^]^ resulting in their being perceived as morally incomplete or non‐moral entities.^[^ ^13^, ^93^, ^96^, ^97^, ^98^ ^]^ Consequently, moral expectations, responsibility attributions, and evaluative standards applied to AI agents differ markedly from those for humans.^[^ ^8^, ^93^, ^99^ ^]^ These divergences span interpersonal, societal, and even religious dimensions,^[^ ^92^, ^99^, ^100^ ^]^ underscoring the need for further empirical and theoretical study of moral judgments involving non‐human agents like AI.

Contemporary moral psychologists emphasize moral pluralism, exploring moral issues across multiple domains.^[^ ^80^, ^101^, ^102^ ^]^ Within this framework, the Moral Foundation Theory (MFT) serves as a pivotal model for understanding human moral preferences.^[^ ^103^ ^]^ This theory proposes that humans have evolved five distinct foundational moral preferences to navigate adaptive challenges associated with out‐of‐group threats,^[^ ^80^, ^104^, ^105^ ^]^ including concern for others' suffering (Harm/Care), proportional fairness (Fairness/Reciprocity), group loyalty (Ingroup/Loyalty), respect for authority and tradition (Authority/Respect), and concerns with purity and contamination (Purity/Sanctity).^[^ ^106^, ^107^ ^]^ Research has shown that moral preferences have a significant genetic basis.^[^ ^108^ ^]^ For example, Bernhard et al.^[^ ^109^ ^]^ found that polymorphisms in specific genes, such as the oxytocin receptor gene, can significantly influence complex moral decision‐making processes, providing direct evidence for the genetic basis of moral preferences. Animal studies further support this view: observations of chimpanzee social behaviors, such as food sharing, reveal that they exhibit moral preferences akin to the principle of reciprocity.^[^ ^110^ ^]^ These zoological findings suggest that moral preferences may have been conserved through evolution and possess a genetic basis in both humans and other species.^[^ ^111^ ^]^

At the social cognition and behavioral level, individuals who prefer loyalty and authority are more likely to hold negative attitudes toward individuals or groups that challenge or threaten existing authority structures.^[^ ^112^ ^]^ Additionally, individuals who prioritize fairness are also more inclined to endorse policies that restrict the rights of out‐group members.^[^ ^113^ ^]^ In recent years, this phenomenon has gained attention in AI research. For example, when AI agents disobey organizational commands that strongly emphasize authority principles (such as in the military or government), individuals with a moral preference for authority are more likely to interpret such actions as defiance and respond negatively.^[^ ^99^, ^114^, ^115^ ^]^ Additionally, studies on religious authoritarian beliefs and robots’ anxiety have found that individuals with a moral preference for sanctity tend to have more negative views of robots.^[^ ^116^ ^]^ Based on this, the present study hypothesizes that moral preferences mediate negative attitudes toward AI agents, meaning that individuals with higher moral preferences are more likely to hold negative attitudes toward AI agents, especially when AI agents are perceived as a potential threat to the established social order.

## 3 Hypothesis Development

This study aims to explore the genetic mechanisms underlying negative attitudes toward AI agents and related personality traits from a behavioral genetics’ perspective. Using twin analysis, we assessed 162 twin pairs using a self‐report scale aligned with the research questions of this study, aiming to measure relevant psychological variables.

**Figure 1:**
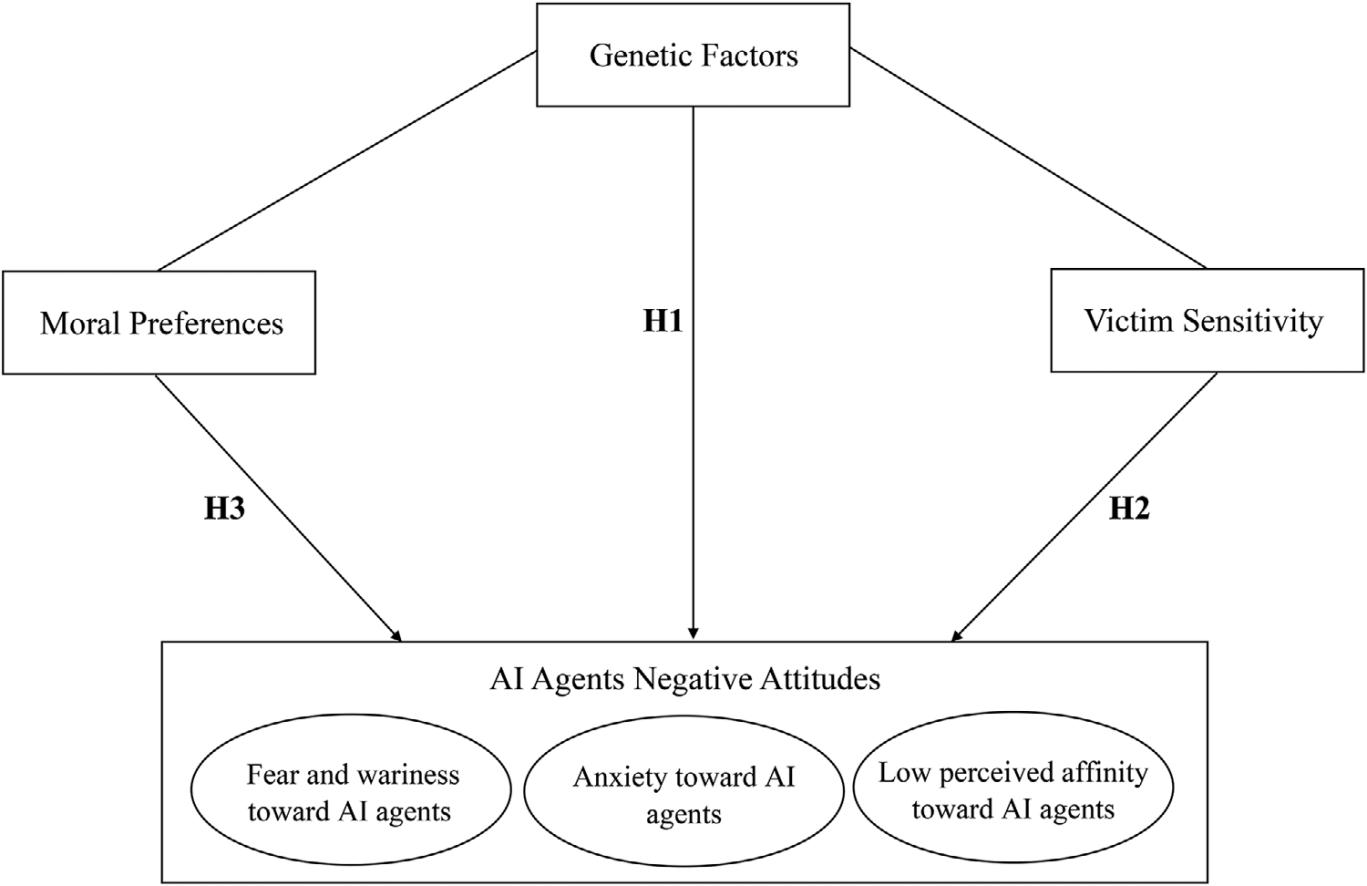
Research model.

Based on the literature (see above), we propose the following hypotheses (see **Figure** **1**):

**Hypothesis 1**. Negative attitudes toward AI agents are heritable, that is, their individual differences are partially explained by genetic factors.

**Hypothesis 2**. The correlation between victim sensitivity and negative attitudes toward AI agents is partly due to genetic factors.

**Hypothesis 3**. The correlation between moral preferences and negative attitudes toward AI agents is partly due to genetic factors.

## 4 Methods

### 4.1 Participants

This study was preregistered on the Open Science Framework (https://osf.io/jvpcz). The full set of dependent measures reported in this article was completed by a total of 164 Chinese twin‐pairs (*n* = 328) sampled from the Beijing Twin Study (BeTwiSt), consisting of 98 pairs of monozygotic (MZ) twins and 66 pairs of same‐sex dizygotic (DZ) twins. Two DZ twin pairs were excluded from the analysis during data screening due to highly similar response patterns across all the items. Consequently, the final sample consisted of 162 twin pairs (*n* = 324; age range: 25–35 years, mean ± standard deviation[*SD*] = 29.90 ± 2.50 years), with 98 MZ pairs (51 female pairs) and 64 same‐sex DZ pairs (36 female pairs). The BeTwiSt sample is socio‐demographically representative of their counterparts in Beijing, and zygosity was determined through DNA testing, with an accuracy rate near 100%.^[^ ^117^ ^]^ The study was approved by the Ethics Committee of Institute of Psychology, Chinese Academy of Sciences, with the approval number H25016. All procedures involving human participants were conducted in accordance with the ethical standards of the committee and with the 1964 Helsinki declaration and its later amendments or comparable ethical standards. Informed consent was obtained from all participants before conducting the study, in accordance with the ethical guidelines.

### 4.2 Measures

Prior to completing the questionnaires, participants were first introduced to the concept of AI agents. They were provided with a definition of AI agents and informed that AI agents encompass both physical forms (e.g., robots) and virtual forms (e.g., voice assistants). Additionally, illustrations sourced from the internet were presented for each type of AI agent. Then, all participants proceeded to complete the following questionnaires.

#### 4.2.1 Questionnaires on Negative Attitudes toward AI Agents

*General Attitudes Toward Robots Scale (GAToRS)*. Participants’ fear and wariness toward robots was measured using the GAToRS.^[^ ^55^ ^]^ This multi‐doman scale was developed to assess individuals’ attitudes toward robots and differentiated negative attitudes into two levels: (1) Personal‐level negative attitude (P‐): unease and anxiety around robots (e.g., “Robots scare me.”); and (2) Societal‐level negative (S‐): rational worries about robots in general (e.g., “Robots may make us even lazier.”). Both subscales demonstrated acceptable internal consistency (Cronbach’ s *α* = .70 and .75, respectively). Participants rated statements on a 7‐point Likert scale (1 = strongly disagree, 7 = strongly agree). We computed the mean score for each subscale by averaging the responses to the respective items.

*Artificial Intelligence Anxiety Scale (AIAS)*. The AIAS^[^ ^61^ ^]^ is a 21‐item self‐report scale designed to assess individuals’ anxiety levels toward AI. It consists of four factors: (1) learning (e.g., “Learning to use AI techniques/products makes me anxious.”; *α* = .96); (2) job replacement (e.g., “I am afraid that AI techniques/products will replace someone's job.”; *α* = .91); (3) sociotechnical blindness (e.g., “I am afraid that an AI technique/product may lead to robot autonomy.”; *α* = .92); and (4) AI configuration (e.g., “I find humanoid AI techniques/products (e.g., humanoid robots) scary.”; *α* = .94). All items are scored on a 7‐point Likert scale (1 = strongly disagree, 7 = strongly agree). We computed the respective scores by averaging the items for each factor.

*Affinity Toward robots*. Participants’ affinity toward robots was measured using the familiarity dimension of the Multi‐dimensional Robots Attitude Scale^[^ ^118^ ^]^ (Some researchers translate “affinity” as “familiarity.”^[^ ^17^ ^]^), which includes five items (*α* = .87), such as “I would feel relaxed with a robot in my home.” Participants indicated the extent to which each item reflected their feelings or thoughts about robots on a 7‐point Likert scale (1 = not at all, 7 = very much). The average score across items was used as an index of affinity toward robots.

#### 4.2.2 Personality Trait‐Related Questionnaires

*Justice Sensitivity Inventory (JSI)*. Participants completed victim sensitivity subscale of the JSI,^[^ ^83^ ^]^ which measures individuals’ differences in how sensitively they react as victims of injustice. The victim sensitivity scale contains ten items (*α* = .90), such as “It bothers me when others receive something that ought to be mine.” Participants responded to these items using a 6‐point Likert scale (1 = not at all, 6 = extremely). The average score across items was used as an index of victim sensitivity.

*Moral Foundations Questionnaire (MFQ)*. Participants' moral preferences was measured using the MFQ.^[^ ^119^ ^]^ The MFQ was developed to assess individuals' endorsement of the five intuitive moral systems proposed by MFT, with a high internal consistency (Cronbach's *α* = .88). These domains include Harm/Care, Fairness/Reciprocity, Ingroup/Loyalty, Authority/Respect, and Purity/Sanctity. The MFQ comprises two types of items: Relevance Items and Judgment Items. An example of a relevance item from the Authority/Respect domain is: “Whether or not an action caused chaos or disorder”. Participants rated the relevance of moral behaviors using a 6‐point scale (1 = not at all relevant, 6 = extremely relevant) and evaluated their agreement with moral statements on a 6‐point scale (1 = strongly disagree, 6 = strongly agree). Scores for each domain were calculated by averaging all the relevance and judgment items, with higher scores indicating greater moral preference.

### 4.3 Twin Data Analysis

By comparing the resemblance between MZ and DZ twins on observed traits, we can estimate the contributions of additive genetic (A), shared environmental (C), and non‐shared environmental (E) factors to the variance within a trait, as well as the covariance between traits.^[^ ^120^ ^]^ MZ twins are 100% genetically identical, whereas DZ twins are on average 50% identical for additive genetic effects. In the typical cases where twins are raised together, a stronger similarity between MZ twins compared to DZ twins suggests a genetic influence on the trait. The extent to which genetic factors account for variation in a trait or the covariance between traits is known as heritability. Shared environmental components (C) account for familial resemblance through common developmental experiences, while non‐shared environmental factors (E) encompass unique individual experiences and measurement error, collectively explaining residual variance in twin models.

To preliminarily assess genetic and environmental effects on the traits of interest, the intraclass correlation coefficients (ICCs) were calculated to examine the similarity between twins within each zygosity group. For all analyses, participants whose scores exceeding ±3 SD from the sample mean for each trait were excluded. Then, we performed structural equation modeling to partition individual differences (i.e., phenotypic variance) in the traits into three components: additive genetic (A), shared environmental (C), and non‐shared environmental (E) effects. To control for the effects of age and sex, which could inflate twin correlations, we regressed all traits onto these variables and used the standardized residuals hereafter. We implemented the genetic modeling with the R package “OpenMx”^[^ ^121^ ^]^ in the following two stages.

**Figure 2:**
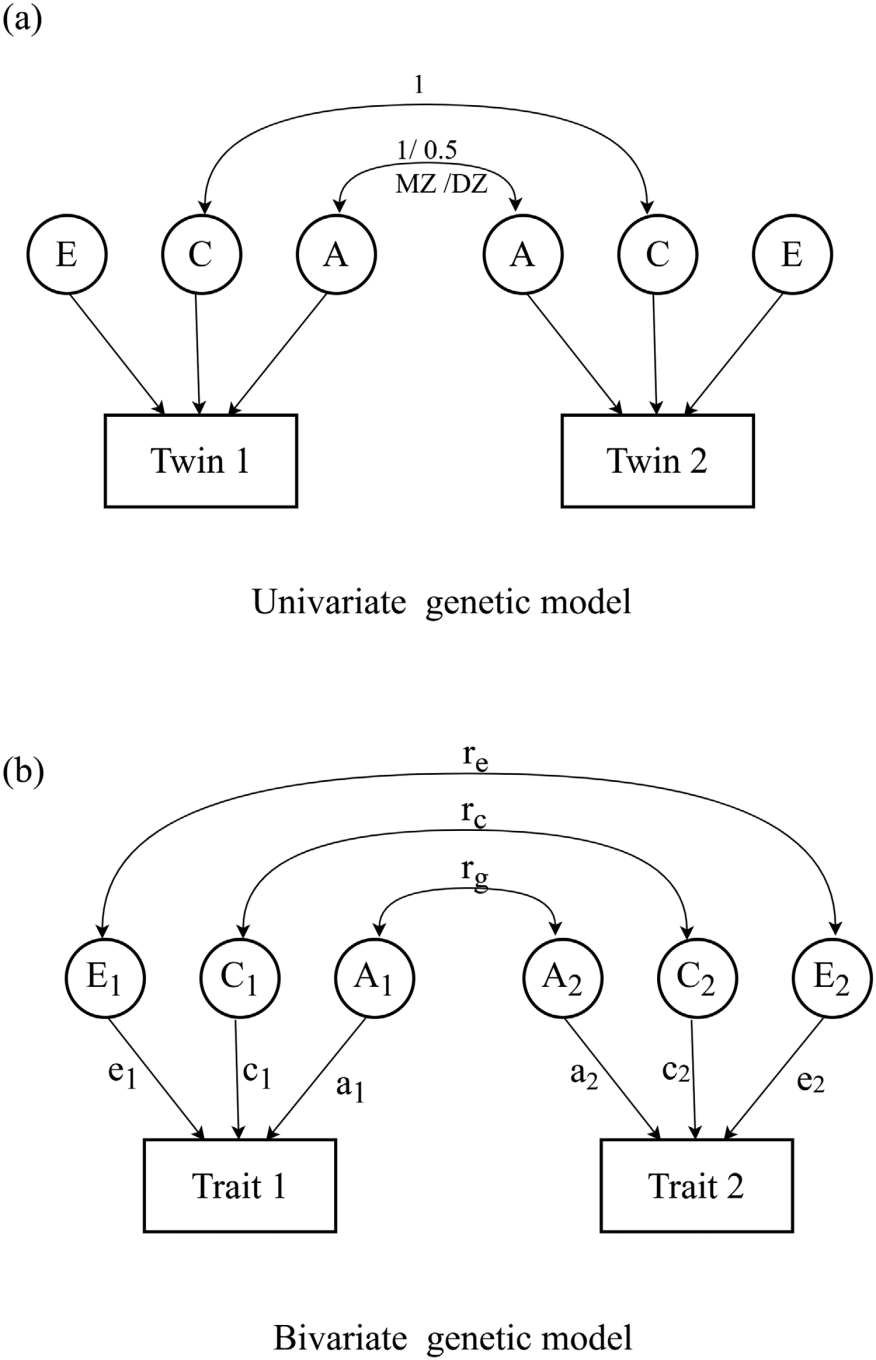
Path diagram illustrating univariate and bivariate genetic model‐fitting. Measured variables are in rectangles. Latent factors A (genetic factors), C (shared environmental factors) and E (non‐shared environmental factors) are in circles. *r_g_*: genetic correlation; *r_c_*: shared environmental correlation; *r_e_*: non‐shared environmental correlation.

First, we applied univariate ACE models to all the traits of interest separately, to estimate genetic (A) and environmental (C and E) effects (see **Figure** **2**A). In accordance with classical twin modeling conventions,^[^ ^120^, ^122^, ^123^ ^]^ genetic factors were modeled as correlated at 1.00 for MZ twin pairs and 0.50 for DZ twin pairs, reflecting their known differences in genetic relatedness. Under the equal environments assumption (both MZ and DZ twins are assumed to share environmental influences to the same extent when raised in the same family), shared environmental (C) factors were correlated at 1.00 for both twin types. Non‐shared environmental (E) components, capturing individual‐specific experiences and measurement error, were assumed to be uncorrelated between the two siblings. For each trait, the full ACE model was examined first, followed by sub‐models (AE, CE, and E) where one or two components were systematically removed.

Next, we conducted bivariate genetic analyses using a correlated factors model to assess how genetic and environmental sources contributed to the phenotypic relationship between AI attitudes and other psychological processes^[^ ^124^ ^]^ (see Figure 2B). In the bivariate model, each trait is separately decomposed into ACE components; meanwhile, their phenotypic correlation was decomposed into genetic (*r*_g_), shared environmental (*r*_c_), and nonshared environmental components (*r*_e_). A significant *r_g_* indicates that the genetic effects on both traits share some overlap. As in the univariate analysis, the full ACE model and all of the sub‐models (AE, CE and E) were systematically tested. To ensure the bivariate genetic modeling was meaningful, we performed such analyses only for those genetically influenced and correlated traits (e.g., personal‐level negative attitudes toward robots and victim sensitivity).

Model fit was evaluated using the change in chi‐square (χ^ 2^) and the Bayesian Information Criterion (BIC).^[^ ^125^ ^]^ A lower BIC value indicates a better fit. When comparing the full model to a sub‐model, a significant chi‐square difference suggests that the sub‐model fits significantly worse, in which case the full model should be preferred; otherwise, the sub‐model with fewer parameters should be considered in terms of parsimony.^[^ ^126^ ^]^ To increase statistical power of genetic model‐fitting, all available data were included even if the data from several twin pairs were not pairwised.

### 4.4 Statistical Analysis

Data preprocessing included outlier removal and assessment of distributional assumptions. Outliers were defined as values exceeding ± 3 SD from the mean for each variable and were excluded. The univariate normality of continuous variables was assessed using Q–Q plots and Kolmogorov–Smirnov tests (*α* = 0.05). As no substantial deviations from normality were observed, variables were analyzed in their original scale and are reported as mean ± SD.

Primary analyses utilized the full sample (*N* = 324; 162 twin pairs). ICCs were calculated separately for MZ (*n* = 98 pairs) and DZ (*n* = 64 pairs) twins, with between‐group differences tested using one‐tailed Fisher's z tests (H_1_: *r*_MZ_ > *r*_DZ_, *α* = 0.05). Phenotypic correlations were examined using Pearson's r, with two‐tailed *p*‐values adjusted for multiple comparisons via false discovery rate (FDR, *q* < 0.05). For genetic modeling, continuous variables were residualized for age and sex effects through linear regression, and standardized residuals were used as inputs. Nested model comparisons employed likelihood ratio tests (*χ* ^2 ^difference tests, *α* = 0.05).

All statistical analyses were performed in R version 4.4.1 (OpenMx 2.21.13 for structural equation modeling; https://cran.r‐project.org/bin/windows/base/old/4.4.1) and SPSS version 28 (https://www.ibm.com/cn‐zh/spss).

## 5 Results

### 5.1 Descriptive Statistics

Descriptive statistics for all measures for MZ and DZ pairs are presented in **Table** **1**.

**Table 1:** Descriptive statistics for all measures.

Measures	N	Min	Max	Mean	SD
GAToRS					
P‐	323	1.00	5.25	3.07	0.86
S‐	324	2.80	7.00	5.50	0.88
AIAS					
Learning	324	1.00	6.13	3.08	1.19
Job replacement	324	1.00	7.00	4.80	1.27
Sociotechnical blindness	324	1.00	7.00	4.94	1.27
AI configuration	324	1.00	7.00	3.71	1.53
Affinity toward robots	324	1.40	7.00	4.74	1.07
JSI_Victim	323	1.00	4.40	2.54	0.72
MFQ					
Harm/Care	321	2.50	5.67	4.25	0.60
Fairness/Reciprocity	323	2.33	5.67	4.24	0.62
Ingroup/Loyalty	323	2.50	5.83	4.47	0.64
Authority/Respect	323	2.50	6.00	4.13	0.66
Purity/Sanctity	324	2.00	6.00	3.77	0.68

N: number of twins. GAToRS (P‐): personal‐level negative attitude toward robots; GAToRS (S‐): societal‐level negative attitude toward robots; AIAS: AI Anxiety Scale; JSI_Victim: victim sensitivity; MFQ: Moral Foundations Questionnaire. For each measure, participants whose scores exceeded ±3 SD from the sample mean were excluded.

### 5.2 Twin Correlations

ICCs for all measures are presented in **Table** **2**. For societal‐level negative attitude toward robots (*p* = .006), sociotechnical blindness toward AI agents (*p* < .001), affinity toward robots (*p* < .001), victim sensitivity (*p* = .049), and moral preferences concerning authority and purity (*p*s < .001), MZ twins demonstrated substantially higher intraclass correlations than DZ twins across these measures (one‐tailed Fisher's *z* tests), providing evidence for additive genetic effects. Accordingly, we proceeded with univariate genetic analyses of these traits. For personal‐level negative attitude toward robots, although MZ twins showed higher intraclass correlations than DZ twins, this difference was not significant (*p* = .116). Given the consistent pattern of higher MZ correlations across the other negative attitude‐related measures, we proceeded to fit genetic models for this trait to explore its potential genetic contribution. In contrast, for the remaining three domains of AI anxiety (learning anxiety, job replacement, and AI configuration) and three domains of the MFQ (Harm/Care, Fairness/Reciprocity, Ingroup/Loyalty), MZ twins did not show significantly higher intraclass correlations than DZ twins. Therefore, genetic models were not fitted for these traits.

**Table 2:** Twin intraclass correlations for all measures.

Measures		Twin correlations					
	ICC_MZ_		95%CI	N_MZ_	ICC_DZ_	95%CI	N_DZ_
GAToRS							
P‐	.42^**^		.14‐.61	98	.30	−.16–.58	63
S‐	.49^***^		.24‐.66	98	.24	−.25‐.54	64
AIAS							
Learning	−.05		−.55‐.30	98	.23	−.25‐.53	64
Job replacement	.25		−.13‐.50	98	.16	−.39‐.49	64
Sociotechnical blindness	.43^**^		.15‐.62	98	.09	−.51‐.45	64
AI configuration	.23		−.15‐.49	98	.37^*^	−.05‐.61	64
Affinity toward robots	.67^***^		.51‐.78	98	.38^*^	−.03‐.63	64
JSI_Victim	.58^***^		.37‐.72	97	.44^*^	.08‐.66	64
MFQ							
Harm/Care	−.09		−.63‐.27	96	.03	−.60‐.41	63
Fairness/Reciprocity	−.002		−.51‐.33	97	−.07	−.76‐.35	64
Ingroup/Loyalty	.27		−.09‐.51	97	−.07	−.74‐.35	64
Authority/Respect	.42^**^		.13‐.61	98	−.01	−.65‐.38	63
Purity/Sanctity	.40^**^		.10‐.60	98	−.08	−.72‐.34	64

ICC: intraclass correlation; MZ: monozygotic twins; DZ: dizygotic twins; 95% CI: 95% confidence interval; N: number of twin pairs; GAToRS (P‐): personal‐level negative attitude toward robots; GAToRS (S‐): societal‐level negative attitude toward robots; AIAS: AI Anxiety Scale; JSI_Victim: victim sensitivity; MFQ: Moral Foundations Questionnaire. For each measure, participants whose scores exceeded ±3 SD from the sample mean were excluded.

### 5.3 Univariate Model‐Fitting

**Table** **3** displays the results of model‐fitting and parameter estimates of the univariate models for personal‐ and societal‐level negative attitudes toward robots, sociotechnical blindness anxiety toward AI agents, affinity toward robots, victim sensitivity, and moral preferences concerning authority and purity.

**Table 3:** Univariate genetic model‐fitting.

Measures	Model				Change from full model			Parameter estimates		
		−2LL	*df*	BIC	Δ*χ* ^2^	Δ*df*	*P*	a^2^	c^2^	e^2^
GAToRS										
P‐	ACE	906.99	319	−715.95				.16(.00–.43)	.10(.00–.37)	.74(.57–.91)
	**AE**	907.13	320	−720.90	0.14	1	0.704	.27(.10–.43)		.73(.57–.90)
	CE	907.28	320	−720.75	0.29	1	0.593		.23(.08–.37)	.77(.63–.92)
	E	916.10	321	−717.02	9.11	2	0.011			1.00
S‐	ACE	906.32	320	−721.71				.32(.00–.47)	0(.00–.37)	.68(.53–.82)
	**AE**	906.32	321	−726.80	0.00	1	1.000	.32(.15–.47)		.68(.53–.85)
	CE	907.54	321	−725.58	1.22	1	0.269		.26(.24–.40)	.74(.60–.76)
	E	918.67	322	−719.54	12.35	2	0.002			1.00
Sociotechnical Blindness Anxiety	ACE	911.67	320	−716.36				.28(.00–.46)	0(.00–.27)	.72(.54–.85)
	**AE**	911.67	321	−721.45	0.00	1	1.000	.28(.09–.46)		.72(.54–.91)
	CE	914.29	321	−718.82	2.62	1	0.105		.18(.03–.33)	.82(.67–.97)
	E	919.84	322	−718.37	8.16	2	0.017			1.00
Affinity Toward Robots	ACE	887.95	320	−740.08				.49(.04–.61)	0(.00–.42)	.51(.39–.65)
	**AE**	887.95	321	−745.17	0.00	1	1.000	.49(.33–.61)		.51(.39–.67)
	CE	891.67	321	−741.44	3.73	1	0.054		.39(.27–.51)	.61(.49–.73)
	E	918.51	322	−719.69	30.6	2	< 0.001			1.00
JSI_Victim	ACE	892.58	319	−730.37				.31(.00–.57)	.12(.00–.47)	.57(.43–.74)
	**AE**	892.84	320	−735.19	0.26	1	0.610	.44(.29–.57)		.56(.43–.71)
	CE	893.95	320	−734.08	1.38	1	0.240		.37(.31–.49)	.63(.51–.69)
	E	917.03	321	−716.09	24.45	2	< 0.001			1.00
MFQ_Authority	ACE	910.07	319	−712.88				.23(.04–.40)	0(.00–.25)	.77(.60–.96)
	**AE**	910.07	320	−717.97	0.00	1	1.000	.23(.04–.40)		.77(.60–.96)
	CE	912.16	320	−715.87	2.09	1	0.148		.15(.00–.29)	.85(.71–1.00)
	E	915.75	321	−717.37	5.68	2	0.058			1.00
MFQ_Purity	ACE	914.99	320	−713.04				.19(.04–.37)	0(.00–.23)	.81(.63–.99)
	AE	914.99	321	−718.13	0.00	1	1.000	.19(.01–.37)		.81(.63–.99)
	CE	916.83	321	−716.29	1.84	1	0.175		.12(.00–.27)	.88(.73–1.00)
	**E**	919.21	322	−719.00	4.22	2	0.121			1.00

−2LL: twice the negative log‐likelihood; BIC: Bayesian Information Criterion;Δ*χ*2: change in chi‐square;Δ*df*: change in degrees of freedom (*df*); a^2^: proportion of variance due to additive genetic effects (A); c^2^: proportion of variance due to shared environmental effects (C); *e^2^*: proportion of variance due to non‐shared environmental effects (E); 95% confidence intervals are reported in parentheses. E, CE, and AE models are nested within the ACE model. The best‐fitting models are shown in bold. GAToRS (P‐): personal‐level negative attitude toward robots; GAToRS (S‐): societal‐level negative attitude toward robots; JSI_Victim: victim sensitivity; MFQ_Authority: moral preferences concerning authority; MFQ_Purity: moral preferences concerning purity. For each measure, participants whose scores exceeded ±3 *SD* from the sample mean were excluded.

#### 5.3.1 Negative Attitudes Toward Robots

*Personal‐ and Societal‐Level Negative Attitudes Toward Robots*. According to the parsimony principle,^[^ ^126^ ^]^ the AE models were more desirable for both personal‐ (P‐) and societal‐level (S‐) negative attitudes, as removal of shared environmental (C) components did not significantly compromise model fit (Δ*χ* ^2^s ≤ .14,  *p*s≥.704). In the best‐fitting AE models, additive genetic factors accounted for 27% of variance in personal‐level negative attitude and 32% of variance in societal‐level negative attitude toward robots, with non‐shared environmental factors explaining the residual variance (73% and 68%, respectively).

*Anxiety Toward AI Agents*. For sociotechnical blindness anxiety toward AI agents, the best‐fitting model was also the AE model, where the additive genetic component accounted for 28% of the variance, and the non‐shared environment accounted for 72% of the variance.

*Affinity Toward robots*. The full ACE model identified 49% of individual difference in affinity toward robots due to genetic influences and the other 51% attributed to non‐shared environmental influences, with zero contribution from shared environments. Hence, the AE model (excluding C) fitted the data as well as the full model (Δ*χ* ^2^ = 0.00, *p* = 1.00).

#### 5.3.2 Personality Traits

As **Table** **3** shows, the parsimonious AE model provided optimal fit for both victim sensitivity and moral preferences concerning authority. Additive genetic factors accounted for 44% of variance in victim sensitivity and 23% in moral preferences concerning authority, while non‐shared environment accounting for 56% and 77%, respectively. In contrast, for the moral preferences concerning purity, the E model offered the best fit. Removing both additive genetic (A) and shared environmental (C) components did not significantly worsen model fit (Δ*χ* ^2^ = 4.22, *p* = .121), suggesting that genetic factors may not influence the moral preferences concerning purity.

#### 5.3.3 Summary of Univariate Model‐Fitting

Univariate analyses revealed modest to moderate heritability (27–49%) in negative attitudes toward AI agents, with nonshared environmental factors explaining most variance (51–73%). Genetic influences were the strongest for affinity toward robots (49%), followed by societal (32%) and personal level (27%) negative attitudes toward robots, and sociotechnical blindness anxiety toward AI agents (28%). Victim sensitivity (44%) and the moral preferences concerning authority (23%) also showed heritability.

Building on our findings that negative attitudes toward AI agents were heritable, we further examined whether their genetic components overlap with victim sensitivity and moral preferences concerning authority. To investigate this, we first assessed the phenotypic correlations between these constructs, then quantified shared genetic variation using bivariate model‐fitting analyses.

### 5.4 Phenotypic Analyses

**Table** **4** displays the Pearson's correlations among the heritable traits. Personal‐level negative attitude toward robots showed a significant correlation with victim sensitivity (*r* = .15, *p* = .027), while sociotechnical blindness anxiety toward AI agents was significantly associated with moral preferences concerning authority (*r* = .23, *p* < .001). To address potential inflation of correlations due to non‐independence in twin data, we implemented a bootstrap resampling approach (1000 iterations) where one individual was randomly selected per twin pair in each iteration. This approach accounts for the nested structure of the data while generating robust estimates. The resulting correlation estimates, along with bias‐corrected and accelerated (BCa) 95% confidence intervals, are reported in Table 4.

**Table 4:** Phenotypic correlations among all measure.

Measures	1	2	3	4	5	6
GAToRS(P ‐)		.03(−.10, .05)	−.01(−.23, .10)	−.30(−.43, −.26)	.25(.21, .40)	−.03(−.21, .13)
GAToRS(S −)	.16^*^		.14(.10, .22)	.22(.10, .41)	.11(−.06, .24)	.05(−.07, .23)
Sociotechnical Blindness Anxiety	.03	.04		.09(.02, .23)	.15(.11, .27)	.33(.27, .45)
Affinity toward robots	−.20^**^	.17^*^	.00		−.02(−.21, .14)	−.06(−.29, .06)
JSI_Victim	.15^*^	.13^†^	.06	−.01		.04(−.09, .28)
MFQ_Authority	−.03	.01	.23^***^	−.02	−.01	

Lower triangle shows phenotypic correlations based on all participants (N = 324); upper triangle displays bootstrap correlations (1000 clustered resamples of 162 twin pairs) with 95% BCa confidence intervals. Significance levels are adjusted using FDR correction: ^***^ *p* ≤ .001, ^**^ *p* ≤ .01, ^*^ *p* ≤ .05, † indicates marginal significance. GAToRS(P‐): personal‐level negative attitude toward robots; GAToRS(S‐): societal‐level negative attitude toward robots; JSI_Victim: victim sensitivity; MFQ_Authority: moral preferences concerning authority.

### 5.5 Bivariate Model‐Fitting

**Figure 3:**
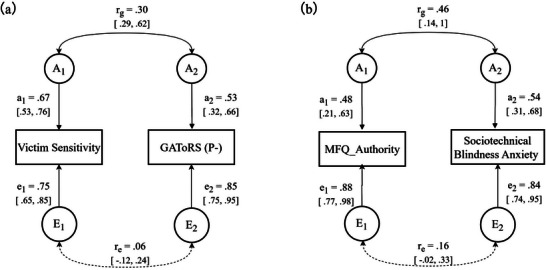
The best‐fitting bivariate genetic models: a) the best‐fitting model for Victim Sensitivity and GAToRS (P‐); b) the best‐fitting model for MFQ_Authority and Sociotechnical Blindness Anxiety. Measured variables are in rectangles. Latent factors A (additive genetic factors) and E (non‐shared environmental factors) are in circles. *r_g_*: genetic correlation; *r_e_*: non‐shared environmental correlation. All path estimates (95% confidence intervals), standardized but unsquared, are obtained from the best‐fitting model. GAToRS (P‐): personal level negative attitude toward robots; MFQ_Authority: moral preferences concerning authority.

Building on these findings, we employed correlated factors models to investigate genetic and environmental influences on the associations between different traits: one for personal‐level negative attitude toward robots and victim sensitivity, and the other for sociotechnical blindness anxiety toward AI agents and moral preferences concerning authority (see **Table** **5** and **Figure** **3**).

**Table 5:** Bivariate genetic model‐fitting.

Model	−2LL	*df*	BIC	Change from full model		
				Δ*χ*2	Δ*df*	*p*
Victim Sensitivity – GAToRS(P‐) (Figure 2a)						
ACE	1792.75	635	−1437.87			
**AE**	1793.53	638	−1452.36	.78	3	.85
CE	1795.16	638	−1450.73	2.41	3	.49
E	1826.61	641	−1434.54	33.86	6	0
MFQ_Authority – Sociotechnical Blindness Anxiety (Figure 2b)						
ACE	1803.68	636	−1432.03			
**AE**	1803.6 8	639	−1447.30	0	3	1
CE	1812.14	639	−1438.84	8.46	3	.04
E	1816.98	642	−1449.25	13.31	6	.04

‐2LL: twice the negative log‐likelihood; BIC: Bayesian Information Criterion; Δ*χ*2: change in chi‐square; Δ*df*: change in degrees of freedom (df); A: additive genetic effects; C: shared environmental effects; E: non‐shared environmental effects. E, CE, and AE models are nested within the ACE model. The best‐fitting model shown in bold. GAToRS(P‐): personal‐level negative attitude toward robots; MFQ_Authority: moral preferences concerning authority.

#### 5.5.1 Personal‐Level Negative Attitude Toward Robots and Victim Sensitivity

In line with the results of the univariate analyses, the AE models was optimal for personal‐level negative attitude toward robots and victim sensitivity (see Table 5). Genes influencing victim sensitivity also moderately influenced GAToRS (P‐) (*r*_g_ = .30, 95% CI[.29, .62]). In contrast, the non‐shared environmental correlation between GAToRS (P‐) and victim sensitivity was nonsignificant (*r*_e_ = .06, 95% CI[‐.12, .24]) (see Figure 3a). The genetic contribution to their phenotypic correlation can be calculated as the product of the genetic path coefficients (from genetic factors to each trait) multiplied by the genetic correlation coefficient, then divided by the phenotypic correlation (i.e., *r*_g_ × *a_1_* × *a_2_* / *r*_p_). Based on model‐estimated parameters, genetic factors accounted for 73.53% of the phenotypic correlation between personal‐level negative attitude toward robots and victim sensitivity, while the non‐shared environment explained the remaining 26.47% of the phenotypic correlation.

#### 5.5.2 Sociotechnical Blindness Anxiety Toward AI Agents and Moral Preferences Concerning Authority

Compared to the full model, the AE model demonstrated an equal fit to the data, whereas the fit of the CE and E models deteriorated significantly. Therefore, the AE model was the best‐fitting model. According to the AE model results, the genetic correlation between AI sociotechnical blindness anxiety and MFQ_Authority was significant *(r_g_* = .46, 95% CI[.14, 1.00]), while the non‐shared environmental correlation was not significant (*r_e_* = .16, 95% CI[–.02, .33]) (see Figure 3b). The association between sociotechnical blindness anxiety toward AI agents and moral preferences concerning authority was explained roughly equally by genetic (50.28%.) and nonshared environmental (49.72%) influences, but only the genetic correlation was significant. To conclude, our bivariate model‐fitting analyses revealed shared genetic influences between personal‐level negative attitude toward robots and victim sensitivity, and between sociotechnical blindness anxiety toward AI agents and moral preferences concerning authority.

## 6 General Discussion and Implications

To our knowledge, this is the first study to reveal the genetic basis of negative attitudes toward AI agents using twin analysis. Our research fills a gap in the literature regarding the genetic foundations of people's negative attitudes toward AI agents. This research gap exists because previous studies on negative attitudes toward AI agents did not consider genetic factors. Our findings reveal the following: First, fear and wariness toward AI agents on a personal level, sociotechnical blindness anxiety toward AI agents (the concern about AI agents losing control and its impacts due to neglecting AI agents' social and technical context), and low perceived affinity toward AI agents show significant heritability (supporting H1). Building on this, we further found that the correlation between the fear and wariness toward AI agents on a personal level and the victim sensitivity is partly due to genetic factors (supporting H2). Additionally, our study indicates that moral preferences concerning authority and sociotechnical blindness anxiety toward AI agents share a common genetic basis (supporting H3).

Through univariate genetic analysis, we found that individuals’ fear and wariness toward AI agents (mainly robots) on a personal level, sociotechnical blindness anxiety toward AI agents, and low perceived affinity toward AI agents (mainly robots) exhibit certain heritability. Meanwhile, environmental influences on these variables primarily stem from non‐shared environmental factors. This suggests that individuals’ negative attitudes toward AI agents are shaped both by genetic predispositions and by unique environmental experiences, such as personal encounters and social interactions. In other words, the differences in individuals' negative attitudes toward AI agents can be partly attributed to each person's unique growth experiences and their ways of perceiving the environment, and partly to genetic factors.

One may wonder why negative attitudes toward AI agents—a novel artificial agents of the technological age—exhibit genetic influences. To further explore potential genetic mechanisms, we conducted bivariate genetic analysis, which revealed that: victim sensitivity and personal‐level fear and wariness toward AI agents (mainly robots) share significant genetic influences. This finding suggests that these psychological traits have a common biological basis at the genetic level. Specifically, an individual's sensitivity and alertness to potential threats may stem from the same genetic mechanisms and manifest in different contexts. To better contextualize these findings, we draw upon TMT, which posits that humans have evolved specialized cognitive and emotional systems for rapidly detecting and responding to potential threats in the environment.^[^ ^127^, ^128^ ^]^ These systems, designed to promote survival, allocate heightened attention and affective resources to stimuli that are novel, ambiguous, or potentially harmful. While shaped in part by cultural and experiential factors, these threat‐detection systems also have a strong genetic basis, contributing to individual differences in vigilance and defensive reactivity.^[^ ^28^ ^]^ Victim sensitivity represents one such individual difference—characterized by a heightened attunement to cues of injustice and personal harm.^[^ ^79^ ^]^ From the perspective of TMT, this trait can be viewed as a manifestation of a hyper‐responsive threat‐monitoring system. This may lead individuals to develop threat amplification biases, in which they overestimate the risks posed by uncertain or novel agents.^[^ ^82^ ^]^ In the case of AI agents, this threat amplification may be especially pronounced. Unlike other nonhuman entities such as animals or mechanical tools,^[^ ^129^, ^130^ ^]^ AI agents possess a hybrid nature that combines features of human‐like agency (e.g., autonomy, decision‐making) with the opacity of algorithmic systems.^[^ ^131^, ^132^, ^133^ ^]^ This combination makes AI uniquely salient: AI systems can exert real‐world influence in socially critical domains such as healthcare, education, and law enforcement, yet their decision‐making processes often remain opaque and difficult to interpret.^[^ ^133^, ^134^, ^135^ ^]^ For individuals high in victim sensitivity—who are particularly attuned to signs of potential exploitation or unfair treatment^[^ ^82^, ^83^ ^]^—the unpredictability and lack of transparency in AI systems may strongly activate defensive threat‐monitoring systems.^[^ ^84^ ^]^ In this sense, AI agents may not only be seen as unfamiliar technologies but also as ambiguous social actors capable of causing harm to the resources and interests related to human communities, thereby eliciting stronger negative appraisals and affective responses compared to other types of nonhuman entities.^[^ ^136^, ^137^ ^]^

At the cognitive level, individuals with high victim sensitivity may exhibit stronger cognitive biases, such as threat amplification or negative attribution bias, when encountering unfamiliar or uncertain technologies.^[^ ^82^, ^138^, ^139^ ^]^ Neuroscientific studies also suggest that individuals with higher victim sensitivity show increased activity in the amygdala and prefrontal cortex when processing potential threats,^[^ ^140^, ^141^ ^]^ regions closely associated with fear and anxiety responses.^[^ ^142^, ^143^ ^]^ While these studies^[^ ^140^, ^141^ ^]^ primarily emphasize the influence of environmental and experiential factors, they nonetheless suggest a plausible neurocognitive pathway through which individual differences in threat processing may operate. Building on this, we propose that the observed shared genetic influences between victim sensitivity and fear or wariness toward AI agents may be expressed, in part, through this threat‐related neurocognitive mechanism—that is, a genetically influenced disposition toward heightened victim sensitivity may shape emotional and cognitive responses via differential activity in the amygdala and prefrontal cortex.

Additionally, our study found that moral preferences concerning authority and sociotechnical blindness anxiety toward AI agents are also influenced by shared genetic factors. This result suggests that these psychological traits may be based on the same genetic mechanisms, reflecting the biological underpinnings of an individual's concern for social order and the stability of power structures. From the perspective of TMT, individuals with strong authority‐oriented moral preferences are particularly sensitive to potential disruptions in social hierarchies.^[^ ^42^, ^80^, ^144^ ^]^ Research indicates that this personality trait is partly influenced by genetic factors and is linked to the neural networks underlying social cognition,^[^ ^41^, ^42^ ^]^ group dynamics, and risk perception, including the medial prefrontal cortex,^[^ ^145^, ^146^ ^]^ anterior cingulate cortex,^[^ ^147^ ^]^ and amygdala.^[^ ^148^, ^149^ ^]^ When AI begins to encroach upon traditionally human‐dominated domains—such as decision‐making, leadership, and moral judgment—it may be perceived as an existential threat to social order, leading to heightened anxiety and resistance.^[^ ^61^, ^62^ ^]^

As AI agents increasingly integrate into social structures, participate in decision‐making, and assume roles of influence, individuals with stronger moral preferences concerning authority may perceive them as symbolic threats—challenges to existing social norms and cultural values—thereby intensifying sociotechnical blindness anxiety toward AI agents.^[^ ^61^, ^62^ ^]^ Sociotechnical blindness anxiety toward AI agents refers to the anxiety caused by neglecting the integration of AI agents with social norms and fearing its widespread adoption may disrupt existing social order and human dominance.^[^ ^60^, ^61^ ^]^ This anxiety may have two key genetic influences. First, individuals with strong moral preferences concerning authority are more inclined to preserve established social hierarchies and exhibit heightened vigilance toward factors that could disrupt these structures.^[^ ^42^, ^150^, ^151^, ^152^ ^]^ Since AI agents possess autonomous decision‐making capabilities and increasingly overlap with human roles in work and social interactions, they may be perceived as “disruptors of order.” Second, sociotechnical blindness anxiety toward AI agents primarily reflects concerns about the potential loss of human control over artificial agents, particularly regarding their autonomous decision‐making abilities.^[^ ^61^ ^]^ Individuals with a strong moral preference concerning authority are generally inclined to maintain the existing social order and remain highly vigilant toward factors that could alter the current power structure.^[^ ^105^, ^153^, ^154^ ^]^ Therefore, when the widespread use of AI agents in society threatens human dominance, these individuals tend to experience stronger anxiety and resistance. This anxiety is not only about technological risks but also represents a deeper resistance to potential changes in social hierarchy, norms, and power distribution.

Therefore, from the perspective of TMT, individuals with a genetic predisposition toward heightened sensitivity to injustice (victim sensitivity) or strong authority‐oriented moral preferences may be more likely to perceive AI agents as potential threats. These cognitive tendencies, in turn, contribute to genetically influenced variations in negative attitudes toward AI. This framework helps explain why some individuals are more resistant to AI integration—not only due to cultural or experiential factors but also because of stable, heritable cognitive and emotional traits that shape their attitudes toward novel socio‐technological entities.

Furthermore, our analysis did not find a significant phenotypic association between moral preferences concerning authority or victim sensitivity and low perceived affinity toward AI agents (mainly robots). Given the absence of a phenotypic relationship, we did not proceed with genetic analyses, as such a foundation is crucial for exploring shared heritability. From a behavioral perspective, the lack of association may stem from differing motivational and psychological underpinnings. For instance, moral preferences related to authority are often driven by concerns about social order, and victim sensitivity is often driven by fairness or personal vulnerability. In contrast, low affinity toward robots may be more closely tied to factors such as social motivation, openness to new experiences, or attitudes toward technology adoption. These distinct motivational frameworks suggest that the two sets of traits are unlikely to share common psychological mechanisms, which could explain their unrelated behavioral manifestations. Moreover, the absence of phenotypic correlation implies that these traits may also lack overlapping genetic or environmental foundations. While shared genetic influences cannot be entirely ruled out, the observed pattern suggests that the genetic and environmental factors shaping moral preferences or victim sensitivity are likely distinct from those influencing attitudes toward robots. It is important to note that our findings should be treated with caution due to the relatively small sample size. Negative results, such as the absence of phenotypic or genetic correlations, may reflect limitations in statistical power rather than definitive evidence of independence. Therefore, future studies should aim to replicate these findings in larger, more diverse populations to confirm the robustness of our conclusions.

Another important question is why only certain dimensions of people's negative attitudes toward AI agents demonstrated heritability, while others—such as job replacement anxiety, configuration anxiety, and learning anxiety (as measured by the AIAS scale)—did not. It is important to note that non‐genetically related dimensions (e.g., learning‐related anxiety, social attitudes, and experience with technology) are not assumed to be entirely free of genetic influence. However, compared to genetically influenced traits, these dimensions are more strongly shaped by individual experience, educational exposure, and sociocultural factors. While evolutionary psychology can offer functional accounts for why such traits emerge, behavioral genetics allows us to determine whether individual differences in these traits are substantially rooted in biological inheritance. Our findings suggest that although various AI‐related anxieties may have adaptive significance, not all are genetically heritable at the individual difference level. Furthermore, only those with significant heritability show genetic overlap with heritable personality traits such as victim sensitivity, suggesting that they may be more deeply anchored in dispositional psychological mechanisms, whereas others are more contingent on environmental variation.

Our study places particular emphasis on the dimensions of negative attitudes toward AI agents that exhibit genetic underpinnings—not to downplay the importance of environmentally influenced factors, but to highlight that such attitudes may not represent a unitary cognitive construct shaped solely by sociocultural factors. Rather, they may consist of heterogeneous components with diverse origins. Identifying genetically influenced dimensions can help reveal that certain negative emotions toward AI agents may reflect deeper, evolutionarily rooted socio‐psychological mechanisms, such as victim sensitivity or concerns for disrupted social order.

### 6.1 Theoretical Contributions

This study, based on a behavioral genetics perspective, reveals the deep connection between attitudes toward AI agents and individual personality traits, expanding the research framework in the field of AI social cognition. First, we found a shared genetic basis between victim sensitivity and fear and wariness toward robots. This suggests that fear and wariness toward robots may partly stem from an individual's inherent threat perception patterns rather than merely uncertainty about new technologies. This finding provides a new theoretical perspective on how individuals assess risks associated with robots and offers an alternative explanation for the formation of fear and wariness toward robots. Second, we found a genetic correlation between moral preferences concerning authority and sociotechnical blindness anxiety toward AI agents. This suggests that there may be a deeper biological basis linking social structure perception and technology acceptance, aligning with the relationship between authoritarianism and attitudes toward social change. This finding opens a new avenue for exploring the biological roots of social cognition in AI perception and highlights the need for a more integrated approach that considers both genetic dispositions and sociocultural influences in shaping technology acceptance.

### 6.2 Practical Implications

These findings have important implications for the social acceptance of AI agents and human‐AI interaction design. First, individual differences in fear and wariness, anxiety, and low perceived affinity toward AI agents are not solely determined by external social factors but are also partially rooted in stable personality traits. Therefore, promoting the societal integration of AI agents through environmental interventions alone (e.g., policy advocacy or technological education) may not be sufficient to eliminate negative public attitudes. Instead, more tailored guidance strategies should be developed for different personality groups. Second, the shared genetic basis between victim sensitivity and fear and wariness toward robots, as well as moral preferences concerning authority and sociotechnical blindness anxiety toward AI agents, suggests that certain individuals may naturally exhibit higher vigilance toward AI agents. This indicates that designers should consider how to reduce the perceived insecurity of these individuals when developing AI agents’ systems. For example, enhancing the predictability and transparency of AI agents could help alleviate psychological burdens and improve user acceptance.

## 7 Limitations, and Future Research

Although this study reveals the genetic and environmental influences on negative attitudes toward AI agents and explores the potential biological basis of related personality traits, several limitations should be noted. First, the study employed the twin‐based behavioral genetics method, which effectively estimates the relative contributions of genetic and environmental factors but does not directly identify specific genetic loci or neurobiological mechanisms. Additionally, the sample size used in this study was relatively small. Future research could integrate genomic analyses (e.g., Genome‐Wide Association Studies) or neuroimaging methods^[^ ^155^ ^]^ and expand the sample size to further investigate how genetic factors shape attitudes toward AI agents. Second, given that negative attitudes toward AI agents may hinder their adoption and technological advancement, it is crucial to investigate the underlying factors shaping such attitudes. Therefore, this study primarily focused on identifying the heritability of negative attitudes toward AI agents. Future research could further investigate whether positive attitudes toward AI agents also have a genetic basis. Additionally, this study found evidence of heritability in anxiety toward AI agents (including both embodied and virtual agents). However, it did not comprehensively consider fear and wariness toward AI agents, as it only employed a scale specifically measuring fear and wariness toward robots. Therefore, future research should further explore the heritability of fear and wariness toward virtual agents. Moreover, this study primarily highlights the role of victim sensitivity and the moral preferences concerning authority. However, other psychological and social factors that may influence negative attitudes toward AI agents—such as social trust and technology acceptance—have not been thoroughly examined. Future research could incorporate multidimensional factors to construct a more comprehensive model of attitude formation toward AI agents, enabling more precise predictions and explanations of individual differences.

Finally, although the current study provides initial evidence for the heritability of attitudes toward AI agents, it remains unclear to what extent these genetic effects are specific to AI‐related evaluations rather than reflecting a general negativity bias. Future research could address this question by including comparative measures of attitudes toward other social entities—such as government institutions, traditional technologies, or human out‐groups—to determine whether AI agents occupies a unique moral and psychological position. Such work would help clarify the domain specificity of genetic influences and enrich our understanding of how humans perceive artificial agents within broader social and moral contexts. Ultimately, understanding individual differences at the biological level will contribute to the development of more personalized AI application strategies, fostering broader social adaptation and integration.

## Conflict of Interest

The authors declare no conflict of interest.

## Author Contributions

X.T. and Y.H. contributed equally to this work as co‐first authors. Y.H. and R.G. designed the research; Y.H. and R.G. contributed to experimental materials; Y.Z., X.L. and Q.D. conducted the experiment and collected the data; X.T. and Y.L. analyzed the data; Y.H., X.T. and R.G. wrote the paper; Y.T., Y.Z. and Y.L. reviewed the paper.
